# *AaCOI1*, Encoding a CORONATINE INSENSITIVE 1-Like Protein of *Artemisia annua* L., Is Involved in Development, Defense, and Anthocyanin Synthesis

**DOI:** 10.3390/genes11020221

**Published:** 2020-02-19

**Authors:** Rong Liu, Jinbiao Wang, Mu Xiao, Xiewang Gao, Jin Chen, Yanjiao Dai

**Affiliations:** 1 Key Laboratory of Plant Development and Environment Adaption, School of Life Sciences, Shandong University, Qingdao 266237, China; lr950310@163.com (R.L.); wangjinbiao@sdu.edu.cn (J.W.); 2 Key Laboratory of Education, Department of Hunan Province on Plant Genetics and Molecular Biology, College of Bioscience and Biotechnology, Hunan Agricultural University, Changsha 410128, China; gaoxiewang@163.com; 3 Institute of Agro-Environment and Ecology, Hunan Academy of Agricultural Sciences, Changsha 410125, China; liuling_66@hnu.edu.cn (J.C.); dyangel_1217@163.com (Y.D.)

**Keywords:** *Artemisia annua*, COI1, jasmonate signaling, development, defense, anthocyanin

## Abstract

*Artemisia annua* is an important medicinal plant producing the majority of the antimalarial compound artemisinin. Jasmonates are potent inducers of artemisinin accumulation in *Artemisisa annua* plants. As the receptor of jasmonates, the F-box protein COI1 is critical to the JA signaling required for plant development, defense, and metabolic homeostasis. *AaCOI1* from *Artemisia annua*, homologous to *Arabidopsis AtCOI1*, encodes a F-box protein located in the nuclei. Expressional profiles of the *AaCOI1* in the root, stem, leaves, and inflorescence was investigated. The mRNA abundance of *AaCOI1* was the highest in inflorescence, followed by in the leaves. Upon mechanical wounding or MeJA treatment, expression of *AaCOI1* was upregulated after 6 h. When ectopically expressed, driven by the native promoter from *Arabidopsis thaliana*, *AaCOI1* could partially complement the JA sensitivity and defense responses, but fully complemented the fertility, and the JA-induced anthocyanin accumulation in a *coi1-16* loss-of-function mutant. Our study identifies the paralog of AtCOI1 in *Artemisia annua*, and revealed its implications in development, hormone signaling, defense, and metabolism. The results provide insight into JA perception in *Artemisia annua*, and pave the way for novel molecular breeding strategies in the canonical herbs to manipulate the anabolism of pharmaceutic compounds on the phytohormonal level.

## 1. Introduction

Jasmonates (JA) refer to a collection of bioactive oxylipins that can be perceived by plants and serve their functions as a fundamental phytohormone with critical roles in plant defense and development. In plants, JA is a family of derivatives from the 16- or 18-carbon tri-unsaturated fatty acids [1]. Jasmonic acid (JA), methyl jasmonate (MeJA), and jasmonoyl-isoleucine (JA-Ile) are well characterized jasmonates, with JA-Ile being the most bioactive form [2]. The metabolic networks and signaling pathway of jasmonates have been a research hotspot over the past decade. To date, the JA-related pathway from JA biosynthesis to JA signaling has been well documented. *LOXs*, *ALLENE OXIDE SYNTHASE* (*AOS*), *ALLENE OXIDE CYCLASE* (*AOC*), and *OXOPHYTODIENOIC ACID REDUCTAXSE 3* (*OPR 3*) are critical genes encoding enzymes required for JA biosynthesis. Meanwhile, COI1, MAPKs, MYC2/3/4/5, JAZs, and ORA59 are reported to play essential roles in the canonical JA signaling pathway [3,4,5].

*CORONATINE INSENSITIVE 1* (*COI1*), encoding a F-box protein, was involved in multiple JA-related processes including stamen development, anthocyanin formation, root growth and wounding response [6,7]. COI1 protein is a part of the Skp1/Cullin/F-box (SCF^COI1^) complex, which has dual E3 ubiquitin ligase activity and JA-Ille-binding activity, and is proposed to be part of the receptor complex of JA-Ile [8]. Upon perception of JA-Ile, COI1 interacts with JASMONATE ZIM DOMAIN (JAZ) proteins and cause their proteasomal degradation, consequently releasing the repression of JAZ-repressed targets, including MYC transcription factors [3,9]. The functions of *COI1* in vascular plants are highly conserved. Even in bryophytes lacking JA-Ile as a ligand, the COI1 proteins are still functional but with altered ligand specificity due to the substitution of a single amino acid residue. Moreover, *COI1* from *Arabidopsis* could rescue the phenotypes of the *Marchantia polymorpha* L. *coi1* mutant, suggesting that the signaling module of COI1 might be ancient and conserved [10]. The soybean *COI1* was able to complement the *Arabidopsis coi1* mutant in fertility and defense responses [11]. The *COI1* gene from *Aquilaria sinensis*, encoding a protein with high similarity to AtCOI1, was strongly induced by mechanical wounding and heat, and its product was located within the nucleus [12]. Overexpression of either *OsCOI1a* or *OsCOI1b* restored JA signaling and production of seeds [13]. *OsCOI1a* and *OsCOI1b* are predominantly involved in the regulation of leaf senescence, and *OsCOI1b* has an additional role in male fertility [14]. The COI1 paralog in tomato, JAI1 is required for male fertility [15]. In hexaploid wheat (*Triticum aestivum* L.), 18 paralogs of COI1 were identified, most of which are highly expressed in the inflorescence or upregulated by stresses [16].

Artemisinin-based combination therapy (ACT) is a prevailing cure for malaria and has saved millions of lives in tropical areas [17]. In recent years, its use in other clinical uses including diabetes and breast cancer has also been proposed [18,19,20,21]. The inadequate supply of artemisinin has prompted extensive studies on artemisinin-related studies. *Artemisia annua* L. is the main source of natural artemisinin. However, the content in plant tissue is very low under natural conditions. A growing body of research has focused on dissection of the metabolic networks [22,23,24,25] and the role of stimulants in artemisinin accumulation [26,27,28,29,30,31,32]. Jasmonate is a potent agent that could increase the yield of artemisinin in *Artemisia annua* plants in several studies [27,29,30,31,32,33,34]. Application of exogenous jasmonate or reconstitution of the JA signaling and modulating the sensitivity to jasmonate through genetic modifications could be reasonable approaches to increase artemisinin accumulation. As COI1 is a conserved module required for JA-signaling in the plant kingdom and the deciphering of *Artemisia annua* genome has provided leverage to launch studies on the COI1 paralogs, which are potential receptors of jasmonates in *Artemisia annua*. In this study, we aimed at characterization of the potential receptor of jasmonates in the canonical medicinal plants *Artemisia annua* L. and provided insight into its roles in JA signaling and JA responses using *Arabidopsis thaliana* as the model.

## 2. Materials and Methods

### 2.1. Plant Materials and Growth Condition

The *A. annua* seeds of variety Huangshuahao 428-A was a gift from Dr. Zhu Weiping (Hunan Agricultural University, Changsha, China). The seeds were sown in autoclaved soil in a 24-cm-diameter pot. Seedlings were grown in a greenhouse at 26 ± 1 °C under 180 μmol photons m^−2^ s^−1^ with a 16/8 h (light/dark) photoperiod before being subjected to assays. The *Arabidopsis* seeds were sowed in autoclaved soil cased in a 7-cm-diameter pot. Seedlings were grown in a growth chamber at 21 ± 1 °C under 120 μmol photons m^−2^ s^−1^ with a 16/8 h (light/dark) photoperiod before being subjected to assays. The *Arabidopsis thaliana* ecotype of Col-0 was used as the wild-type control, and *coi1-16* (CS67817) was ordered from the TAIR and its loss-of-function in *COI1* was validated [35,36]. For the in vitro JA-sensitive assay, the *Arabidopsis* seeds were surface-sterilized with 75% (v/v) ethanol and plated on MS with 50 μM MeJA, followed by two weeks of growth under the abovementioned conditions.

### 2.2. Bioinformatics Analysis

Sequences of *Arabidopsis* COI1 protein (OAP07687.1) was used as queries to search the *Artemisia annua* genome (https://blast.ncbi.nlm.nih.gov/Blast.cgi) with BLAST-P tool. All COI1 proteins (F-box domain) were reconfirmed in SMART (http://smart.embl-heidelberg.de) with the criterion that a F-box domain and at least one LRR domain must be present. Also, the prediction of 3D structure was performed by SWISS-MODEL (https://swissmodel.expasy.org) [37] based on the amino acid sequence of AaCOI1 (PWA88385) (Supplementary Data S1). The 2000 bp upstream of AaCOI1 (PKPP01000819) was designated as the promoter and subjected to analysis by PlantCare (http://bioinformatics.psb.ugent.be/webtools/plantcare/html/) [38,39].

### 2.3. Mechanical Wounding and Chemical Treatments

Thirty-day-old *Artemisia annua* plants were subjected to mechanical wounding and chemical treatment. In detail, clipping the second leaf from the shoot apex by shaving blades was performed to induce mechanical wounding signals, while 50 μM MeJA (Sigma-Aldrich) dissolved in 5% methanol was sprayed onto the second leaf from the shoot apex as the chemical treatment. In *Arabidopsis thaliana*, 50 μM MeJA was sprayed onto the whole plants of three-week-old seedlings [40]. The unwounded plants were set as the control of mechanical treatment, while mock-treatment by 5% methanol was the control of chemical treatment either in *Artemisia annua* or *Arabidopsis thaliana*.

### 2.4. Complementation Assay of AaCOI1 in *Arabidopsis* Coi1 Mutants

The *pFGC5941* binary vector was used as the backbone of the construct for complementation assay. The native promoter of *AtCOI1* (1271 bp) (Supplementary Data S2) was cloned and fused with the coding sequence of *AaCOI*1 (1755 bp) (Supplementary Data S3). The construct was introduced into heterozygous plants of *coi1-16 +/−*. And #1, #2 are segregated from two independent transgenic lines, which are in the *coil-16 −/−*background and harbor the *proAtCOI1::AaCOI1* cassette (Figure S2). All the plants in the background of *coi1-16-/−* are maintained under 16 °C to recover the fertility.

### 2.5. Subcellular Localization of AaCOI1 in *Nicotiana benthamiana*

The pFGC5941 binary vector was used as the backbone of the construct for ectopic expression of *AaCOI1::GFP* cassette driven by the 35S promoter in *Nicotiana benthamiana*. The transient expression in *N. benthamiana* leaves was conducted following the protocol [41,42]. The leaves were sampled and subjected to confocal microscopy (LSM 880, Zeiss™, Jena, Germany) 48 h post-infiltration. 

### 2.6. Bioassay of Herbivory Resistance

Three second-instar larvae of *Spodoptera litura* (Fabricius) were inoculated onto the leaves of four-week-old *Arabidopsis* plants, including wild-type, transgenic and *coi1-*phing and each *16* genotypes. Ten days after infestation, the appearance of infested plants was recorded by photographing. The larvae were weighed to evaluate their growth rate on plants, inversely indicating the resistance of plants.

### 2.7. Analysis of Gene Expression

The total RNA was isolated using the TRIzol reagent (Invitrogen, Carlsbad, CA, USA) following the manufacturer’s instructions. In brief, 2 μg total RNA was reverse-transcribed into cDNA using a RevertAid First Strand cDNA Synthesis kit (Thermo Fisher Scientific, Waltham, MA, USA). The RT-qPCR analysis was performed on a CFX96 Real-Time PCR System C1000 Touch Thermal Cycler (Bio-Rad, Hercules, CA, USA) using the SYBR Green Master mix (Applied Biosystems, Foster City, CA, USA). Two-step qPCR amplification conditions were as follows: 2 min of initial denaturation at 95 °C, 40 cycles at 95 °C for 15 s, 60 °C for 60 s (annealing and extension). The relative expression level of *Artemisia annua* genes and *Arabidopsis thaliana* genes was calculated using the 2^−ΔΔCt^ method [43] using *AaACTIN* (Accession: EU531837.1) and *AtACTIN2* (AT3G18780) as the internal reference genes respectively. The RT-qPCR primers used in the present study are listed in Table S1.

### 2.8. Quantification of MeJA-Induced Anthocyanin

MeJA (50 μM) was applied to induce anthocyanin accumulation in the leaves of *Arabidopsis*. Five days after the initial spray of MeJA, the rosette leaves of Col-0, #1, #2, and coi1-6 plants were collected and subjected to extraction. Equal amount of leaf tissue was grinded into fine powder in liquid nitrogen. The anthocyanin was extracted in solution (Propanol:HCl:H_2_O = 18:1:81) boiled for 3 min and followed by incubation at room temperature in the dark for 4 h. Samples were centrifuged at 12,000 g for 15 min, and the absorbance of the supernatant was measured at 535 and 650 nm by spectrometry. Anthocyanin content was determined by (A535-2.2A650)/g. FW [44].

## 3. Results

### 3.1. Identification of AaCOI1 in *Artemisia annua*

**Figure 1:**
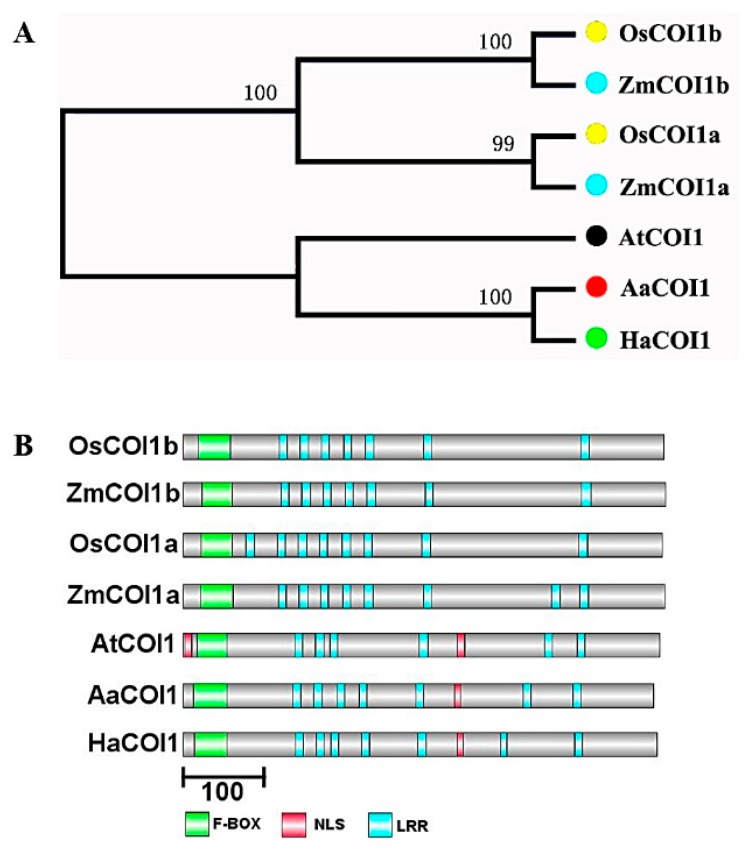
Phylogenetic tree COI1 paralogs (**A**) in *Artemisia annua, Helianthus annuus, Oryza sativa, Zea mays* and *Arabidopsis thaliana,* and their sequences (**B**). The relationships were analyzed for deduced full-length amino acid sequences using MEGA7 by using the Maximum Likelihood (ML) method with Partial deletion and Poisson model, and a Bootstrap test of 1000 replicates for internal branch reliability. Bootstrap values were shown near the nodes. Abbreviations: *Artemisia annua* (Aa)*, Helianthus annuus* (Ha)*, Oryza sativa* (Os)*, Zea mays* (Zm), and *Arabidopsis thaliana* (At).

COI1 paralogs in *Artemisia annua, Helianthus annuus*, *Oryza sativa*, and *Zea mays* were identified by BLAST against the *Arabidopsis thaliana* COI1 protein. The paralogs were selected by the criteria as described in Section 2.2. AaCOI1 were identified in the *Artemisia annua* genome, further termed as AaCOI1 based on the similarity with their *Arabidopsis* homologs (Figure 1). All of the identified COI1 paralogs were clustered (Figure 1A) and their structure was illustrated (Figure 1B). As indicated, AaCOI1 consists of a F-box domain, seven LRR (leucine-rich repeat) domains, and a NLS (nuclear localization sequence) imbedded in the gaps among the LRR sequences.

### 3.2. Sequence Alignment and Structural Analysis

**Figure 2:**
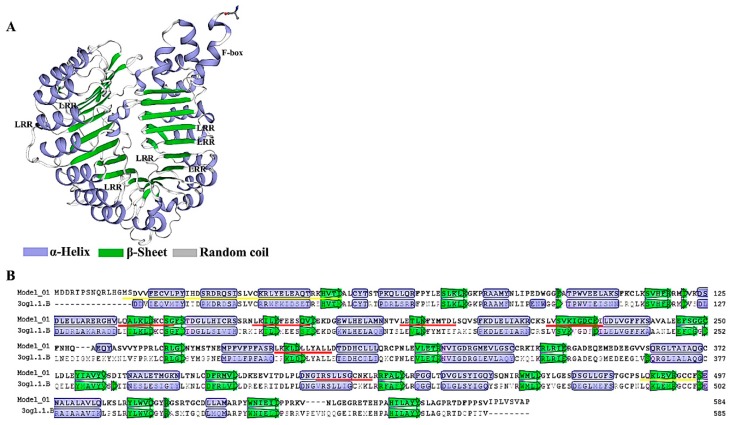
The predicted AaCOI1 3D mode (**A**) and the contribution of amino acid residues to the structure (**B**). The LRRs and F-box are indicated in the figure. The LRRs are marked by red lines, while the F-box is marked by a yellow line (**B**).

A prediction of its 3D structure was conducted by SWISS-MODEL based on its amino acid sequence. Based on the results, the AaCOI1 has an α-helix, a β-sheet, and irregular random coils (Figure 2A). The distribution of amino acid residues in the 3D structure was indicated based on alignment with AtCOI1 sequence (Figure 2B).

### 3.3. Expression Patterns of AaCOI1

**Figure 3:**
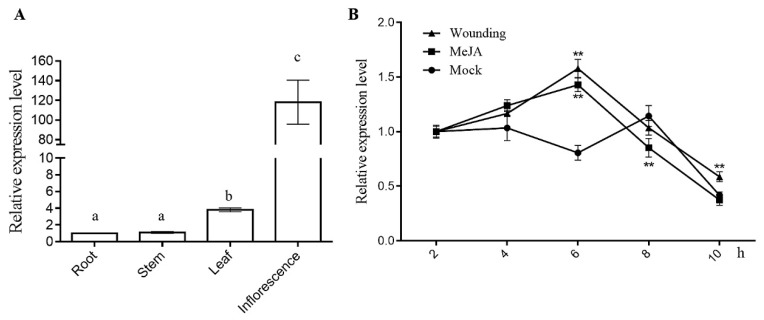
Transcription analysis of *AaCOI* in different tissues (**A**) and in response to wounding and MeJA treatment (**B**). The error bar represents the mean (±standard error) of three independent biological replicates. Columns marked with different letters (**a**–**c**) are significantly different from the others (ANOVA followed by Tukey’s multiple comparison test (*p* < 0.05)); any points of the line chart marked with an asterisk are significantly different (Student’s *t*-test; ** *p* < 0.01). Error bars indicate SD.

In *Arabidopsis*, COI1 functions as a F-box E3-ligase leading to the proteasomal degradation of JAZs and de-repression of JA responses. However, its transcription level was not dramatically regulated by hormones. During various development stages, *COI1* is differentially expressed in root, leaf, shoot, and inflorescence [45]. RT-qPCR was performed to analyze the mRNA abundance of *AaCOI1* in the root, shoot, leaf, and inflorescence of *Artemisia annua*. The results showed *AaCOI1* was highly expressed in leaves and inflorescence (Figure 3A), which carbon-copied the expression pattern of its *Arabidopsis* paralog. However, the expression of *AaCOI1* was dynamically upregulated after 6 h upon MeJA or wounding treatment (Figure 3B).

### 3.4. Subcellular Localization of AaCOI1

**Figure 4:**
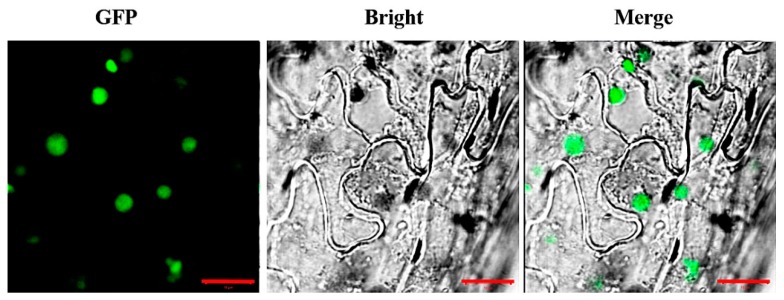
Subcellular localization of AaCOI1. GFP, GFP fluorescence; Bright, bright-field image; Merge, merged GFP and bright-field image. The scale bar represents 10 μm.

To investigate the subcellular localization of AaCOI1, a cassette of *AaCOI1::GFP* fusion driven by 35S promoter was transformed into *N. benthamiana* for transient expression analysis. After 48 h post infiltration, the leaf discs were collected for confocal microscopy. The green florescence protein (GFP) signal was predominantly detected in the nuclei (Figure 4). The GFP without AaCOI1 was also introduced into *N. benthamiana* as the control, and the GFP protein was distributed ubiquitously inside the cells (Figure S1).

### 3.5. Ectopic Expression of AaCOI Partially Rescues Developmental Defects of Coi1-16

**Figure 5:**
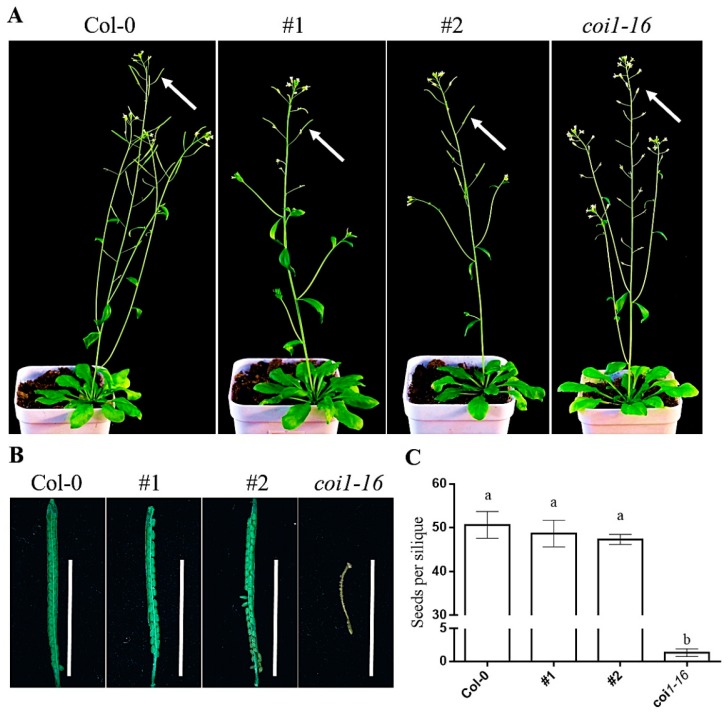
Phenotypes of Col-0, complemented lines #1 and #2, and *coi1-16* mutant. Overall appearance of *Arabidopsis thaliana* plants of different backgrounds (**A**); close-up of fully developed siliques about 10 days after pollination (**B**); the fertility was determined by counting the seeds number per silique (**C**) (*N* > 10). The error bar represents the mean (±standard error) of 10 independent biological replicates. Columns marked with different letters (**a**–**b**) are significantly different from the others (ANOVA followed by Tukey’s multiple comparison test (*p* < 0.05)).

The ectopic expression of the *AaCOI1* coding sequence was driven by the native promoter of *Arabidopsis COI1*. Two independent lines (#1, #2) ectopically expressing *AaCOI1* in the background of *coi1-16* were subjected for further analysis (Figure S2). The *coi1-16* mutant showed abolished fertility resulting in seedless siliques under 22 °C. In the #1 and #2 transgenic lines, the seed number per silique (Figure 5) was higher than that of *coi1-16*, which was similar to wild-type plants, indicating that a successful complementation was imparted by ectopic expression of *AaCOI1*.

### 3.6. Ectopic Expression of AaCOI Restores JA-Sensitivity of Coi1-16

**Figure 6:**
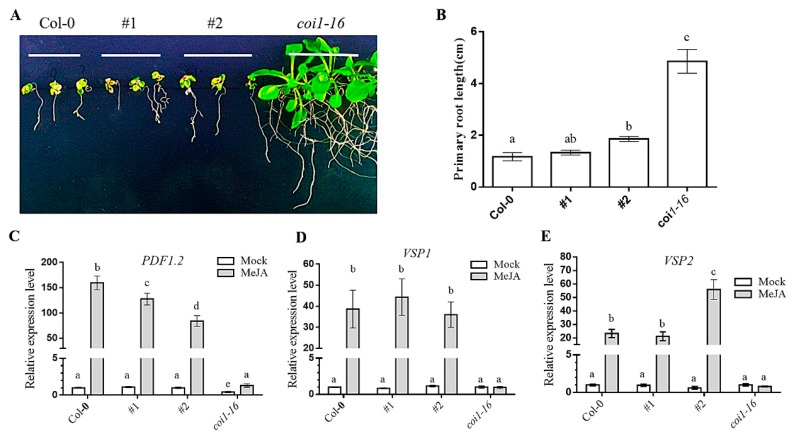
Sensitivity to MeJA of Col-0, complemented lines #1 and #2, and *coi1-16* mutant. Growth of *Arabidopsis thaliana* seedlings on MS supplemented with 50 μM MeJA (**A**); primary root length (N > 10) of seedlings after growing on MS supplemented with 50 μM MeJA for 10 days (**B**); the expression of COI1-dependent and JA-responsive genes in *Arabidopsis* seedlings of different backgrounds ((**C**), *PDF1.2*; (**D**), *VSP1*; (**E**), *VSP2*). The error bar represents the mean (±standard error) of at least three biological replicates. Columns marked with different letters (**a**–**c**) are significantly different from the others (ANOVA followed by Tukey’s multiple comparison test (*p* < 0.05)).

The ectopic expression of the *AaCOI1* coding sequence was driven by the native promoter of *Arabidopsis COI1*. Two independent lines (#1, #2) ectopically expressing *AaCOI1* in the background of *coi1-16* were subjected for further analysis. The *coi1-16* mutant showed normal root growth on MS supplemented with 50 μM MeJA (Figure 6A,B) and impaired induction of *PDF1.2*, *VSP1*, and *VSP2* by MeJA (Figure 6C–E). In contrast, expression of *AaCOI1* in #1 and #2 prioritized their JA sensitivity over that of *coi1-16*, which was indicated by dramatic inhibition of root growth (Figure 6A,B) and significant upregulation of all the three marker genes (Figure 6C–E).

### 3.7. Ectopic Expression of AaCOI1 Imparts Defense against Herbivory in Coi1-16

**Figure 7:**
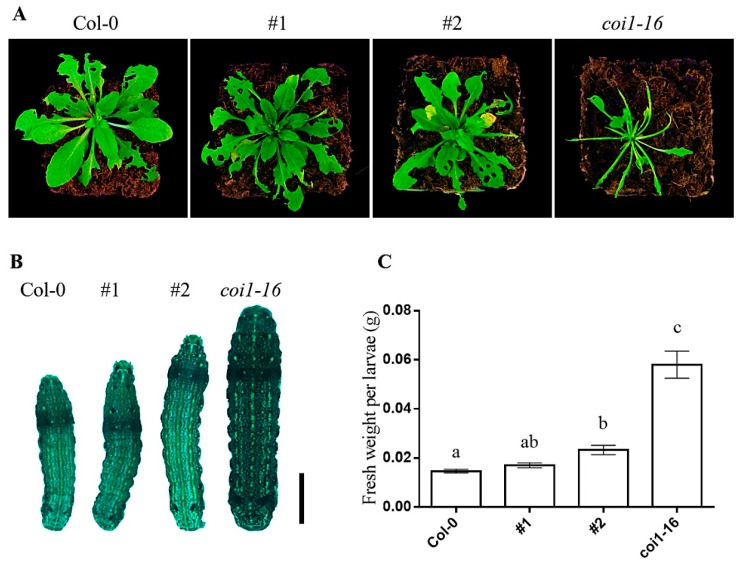
The defense response to herbivory in Col-0, #1, #2, and *coi1-16* mutant. The overall appearance of *Arabidopsis* plants after 10 days of feeding by larvae (**A**); the overall appearance of larvae after feeding on *Arabidopsis* for 10 days (**B**); the fresh weight per larva after 10 days of feeding on *Arabidopsis* plants were measured (**C**). The error bar represents the mean (±standard error) of at least 20 biological replicates. Columns marked with different letters (**a**–**c**) are significantly different from the others (ANOVA followed by Tukey’s multiple comparison test (*p* < 0.05)).

The ectopic expression of the *AaCOI1* coding sequence was driven by the native promoter of *Arabidopsis COI1*. Two independent lines (#1, #2) ectopically expressing *AaCOI1* in the background of *coi1-16* were subjected for further analysis. The *coi1-16* mutant showed attenuated resistance to larval feeding compared to the wild-type plants (Figure 7A), reduced inhibitive effects on larval growth (Figure 7B,C). In the #1 and #2 complement lines, the damage caused by herbivory was similar to that of Col-0 but dramatically reduced compared to that of *coi1-16*.

### 3.8. Ectopic Expression of AaCOI1 Restores the Accumulation of Anthocyanin in Coi1-16

**Figure 8:**
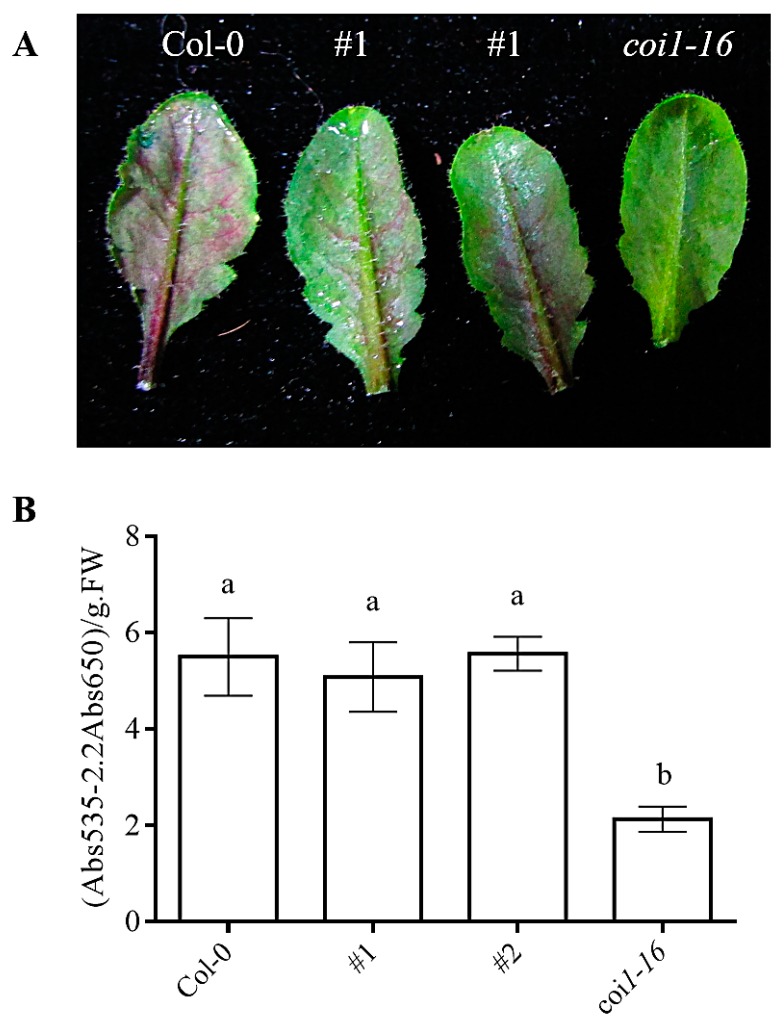
The anthocyanin accumulation in Col-0, #1, #2, and *coi1-16* mutant. The overall appearance of *Arabidopsis* plants after treatment of 50 μM MeJA for 48 h (**A**); the accumulation content measured from 1 g leaf tissue from MeJA-treated Col-0, #1, #2, *coi1-16* (**B**). The error bar represents the mean (±standard error) of at least three biological replicates. Columns marked with different letters (**a**,**b**) are significantly different from the others (ANOVA followed by Tukey’s multiple comparison test (*p* < 0.05)).

The anthocyanin is an important secondary metabolite for its role as a protective flavonoid in planta and a nutritious diet supplement to human, which also have antioxidant activity and radical scavenging potential, anti-aging effects and antidiabetic potential [46,47,48]. JA and COI1 are important in regulation of secondary metabolites including anthocyanins [4,47,49]. The *coil1-16* showed impaired accumulation of anthocyanin upon MeJA treatment. *coi1-16* plants expressing AaCOI1 showed almost the same level of MeJA-induced anthocyanin as the wild-type plants (Figure 8A,B).

## 4. Discussion

Jasmonate-mediated signaling plays multiple roles in plant defense, development, reproduction, and metabolism. Due to its pleiotropic effects, a series of studies have attempted to use jasmonates as a growth regulator to divert the carbon flow to favor the accumulation of the desired metabolites [26,28,29,30,32,33]. Some others have applied genetic engineering to manipulate the JA-governed metabolism [22,23,24,25]. An F-box protein that is encoded by *COI1* is a conserved module in the JA signaling pathway. In *Arabidopsis thaliana*, its role in JA perception and signaling cascade has been extensively studied. Using *Arabidopsis thaliana* as the model, particularly in the background of the loss-of-function mutant *coi1*, a plethora of COI1 homologs have been studied in nonmodel plant species, which further supported the conservation of COI1 and its homologs [10,11,12,13,14,15,16].

Artemisinin is currently the most effective drug so is the basis of ACT therapy. *Artemisia annua* is the main resource of artemisinin, which is not abundant enough for commercial use. The application of jasmonates has great potential to induce the accumulation of artemisinin and tuning of the JA signaling pathway, including the modulation of COI1 activity has also proven potent to regulate plant metabolomics and alter the secondary metabolic profiles [27,29,30,31,32,33,34,50]. In this study, we sought to identify the *Artemisia* orthologue of *AtCOI1*, and characterize its functions in JA-responsiveness, defense and regulation of secondary metabolites.

Based on our phylogenetic analysis, two COI1 homologues were identified in *O. sativa* and *Z. mays*, respectively, termed OsCOI1a/b and ZmCOI1a/b. By contrast, a single copy of COI1 paralog was identified in *Artemisia annua* and in another *Asteraceae* species, *Helianthus annuus*. Furthermore, the AaCOI1 and HaCOI1 were sequestered in the same clade but clearly separated from those in *Arabidopsis thaliana*, *Z. mays*, and *O. sativa* (Figure 1A). Structural analysis revealed all the COI1 paralogs possessed a F-box, and multiple LRR domains (Figure 1B). In detail, ZmCOI1a and OsCOI1a possess eight LRRs each, while ZmCOI1b and OsCOI1b have seven LRRs. AtCOI1 has six LRRs. In *H. annuus*, only six LRRs were identified while there were seven predicted LRRs in *Artemisia annua*. The results strongly suggested the COI1 signaling module had arisen before the separation of monocots and dicots.

In eukaryotes, spatial and temporal transcription of genes is largely dependent on their promoters. Motifs are characteristic cis-elements sequestered on the promoter, which provide binding sites for transcription factors and largely determines the transcription activity of the gene. The 2 kbp upstream of the coding sequence of *AaCOI1* was designated as the promoter (Supplementary Data S4) and subjected to motif prediction by the Plantcare [38,39]. Interestingly, plenty of environment-responsive elements were enriched while few of development-related regulatory elements were identified. Those enriched motifs included MeJA-responsive elements, light-responsive elements, and elements conferring drought inducibility (Table S2). The enrichment of JA-responsive elements might be causative of the responsiveness of AaCOI1 to MeJA and wounding. In addition, the oscillation of AaCOI1 mRNA level in the mock group might be conferred by the light-responsive elements as the total illumination dosage increased in the time course. The *AaCOI1* mRNA abundance were the highest in the inflorescence and *AaCOI1* transcription level was also notably higher in leaves than that in the roots and the stems (Figure 3). Considering the artemisinin content and the expression level of most biosynthesis-related genes are the highest in the flowers and leaves [51], *AaCOI1* expression seems to be positively correlated to artemisinin accumulation, which further supports the role of JA signaling in artemisinin biosynthesis. *AaCOI1* was upregulated by both MeJA and wounding 6 h post-initial treatment, and by wounding 6 h post-initial treatment. To our surprise, at the time point of 9 h after initial exposure to MeJA, the mRNA level of *AaCOI1* was decreased (Figure 3). Based on those results, transcription of *AaCOI1* might be partially responsive to stimuli. In addition, the diverse motifs on the promoter gifted it flexibility in response to the environmental fluctuations.

Based on the 3D structure predicted by SWISS-MODEL [37], the AaCOI1 consisted of α-helix, β-sheet, and the uncharacterized random coil (Figure 2). In *Arabidopsis* and some other species, COI1 is mainly located in the nuclei [12], while the subcellular localization of AaCOI1 remains unknown. The amino acid residue sequence of AaCOI1 contains a predicted NLS (RKIKRLRIE) [52] at the position of 337 amino acid residues from the N-terminus and 7 LRRs [53] (Figure 1B). Transient expression of a fusion cassette of *AaCOI1::GFP* driven by 35S promoter in the *N. benthamiana* was carried out to determine the subcellular localization of AaCOI1. The results showed that AaCOI1 was located in the nuclei (Figure 4). The nuclear localization should be reasonable since COI interacts with JAZ proteins, which are transcription repressors in the nuclei.

To further testify the bona fide functions of *AaCOI1* in plants, an expression cassette of *AaCOI1* driven by the promoter of *AtCOI1* was introduced into the *Arabidopsis coi1-16* mutant, which is glabrous, male sterile, JA-insensitive, and impaired in plant defense responses [35,36]. In two independent transgenic lines (#1, #2), *AaCOI1* complemented the loss-of-function of *Arabidopsis COI1* to different extent (Figure 5, Figure 6 and Figure 7). In detail, ectopic expression of *AaCOI1* in *coi1-16* increased the fertility and seed yield of *coi1-16* (Figure 5). The in vitro growth assay showed the inhibition of root growth in *coi1-16* by MeJA was dramatically relieved by ectopic expression of *AaCOI1* (Figure 6A,B). As JA is a potent elicitor inducing elevation of JA-responsive marker genes (*PDF1.2*, *VSP1*, *VSP2* etc.) [54,55], whether the *coi1-16* mutants expressing *AaCOI1* could respond to MeJA treatment on molecular level was investigated. The results showed that introduction of *AaCOI1* to *coi1-16* restored the JA-induced expression of all the three marker genes (Figure 6C–E). Furthermore, the susceptibility to herbivory of *coi1-16* was almost fully complemented by *AaCOI1*, which was indicated by the leaf damage and larval growth. Interestingly, the capability of complementation of *AaCOI* in *coi1-16* was statistically different. This might be due to the variation of insertion loci resulting different expression level of the introduced *AaCOI1*. Also, we could not exclude the possibility that AaCOI1 protein level was not as high as that of native AtCOI1 in *Arabidopsis* plants as the codon preference varies across species [56].

Anthocyanin is a flavonoid that is involved in plant fertility and stress responses [57,58]. The beneficial effects of anthocyanin to human has also been documented [59,60,61,62]. In *Arabidopsis* and maize (*Z. mays*), JA-signaling promotes the biosynthesis of anthocyanin dependent of *COI1* [47,48,49,63]. To investigate the potential of *AaCOI1* in regulation of JA-induced accumulation of anthocyanin, MeJA was applied to *Arabidopsis* plants in Col-0, #1, #2, or *coi1-16* background respectively. The total content of anthocyanin was measured by spectrometry [43]. Interestingly, both #1 and #2 transgenic lines showed no difference in terms of levels of anthocyanin to the wild-type control, indicating the full complementation of *coi1* by ectopic expression of *AaCOI1*. Unlike the results of complementation assays in development, JA sensitivity, and defense response, basal expression level of *AaCOI1* might be adequate to exert its function in biosynthesis of secondary metabolites. As has been evidenced, upregulated expression level of abscisic acid receptor led to increased artemisinin content [56]. Considering that the anthocyanin is a universal metabolite and COI1-dependent JA-signaling is a conserved pathway in different plant species, our results offered a novel strategy for future genetic improvement in *Artemisia* species to favor the secondary metabolites by tailoring the JA signaling pathway.

## 5. Conclusions

Our study identified *AaCOI1*, which encodes a F-box and LRR-domain containing protein, as a functional paralog of the jasmonate receptor *Arabidopsis COI1*. Expression of *AaCOI1* was responsive to MeJA and wounding. AaCOI1 was localized in the nuclei. In the background of *coi1* null-mutant, ectopic expression of *AaCOI1* driven by *AtCOI1* native promoter partially restored JA sensitivity in root growth and gene induction, and defense to herbivory, but fully complemented the phenotypes in the fertility and JA-induced anthocyanin accumulation. Our results should be insightful by offering a phytohormone-related target for future genetic improvement to modulate development, defense, and total yield in an important medicinal plant, the *Artemisia annua* L.

## Supplementary Materials

The following are available online at https://www.mdpi.com/2073-4425/11/2/221/s1, Figure S1: Subcellular localization of GFP without AaCOI1, Figure S2: Verification of transgenic lines, Table S1: The primers used for constructs and qPCR in the present study, Table S2, Cis-elements identified in the promoter of AaCOI1, Data_S1: Amino acid sequence of AaCOI1, Data S2: Native promoter of AtCOI1, Data S3: AaCOI1 CDS, Data S4: The 2000bp sequence designated as the promoter of AaCOI1.
