# Supplementary material for: AaCOI1, Encoding a CORONATINE INSENSITIVE 1-Like Protein of Artemisia annua L., Is Involved in Development, Defense, and Anthocyanin Synthesis
Source: Genes (Basel). 2020 Feb 19;11(2):221. doi: 10.3390/genes11020221 (PMC7074131; doi:10.3390/genes11020221)
Supplement: Supplementary file 1 [file genes-11-00221-s001.pdf]

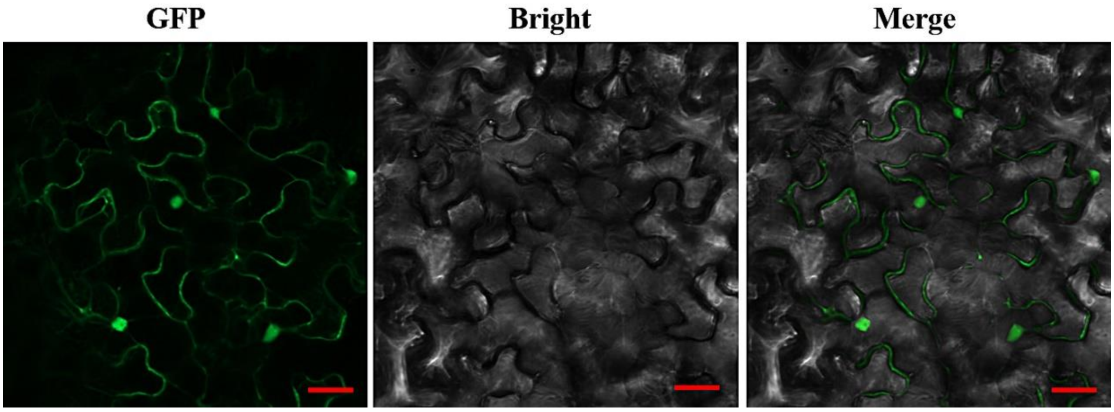

Figure S1. Subcellular localization of GFP without AaCOI1, GFP fluorescence; Bright, bright-field image; Merge, merged GFP and bright-field image. The scale bar represents 10μm.

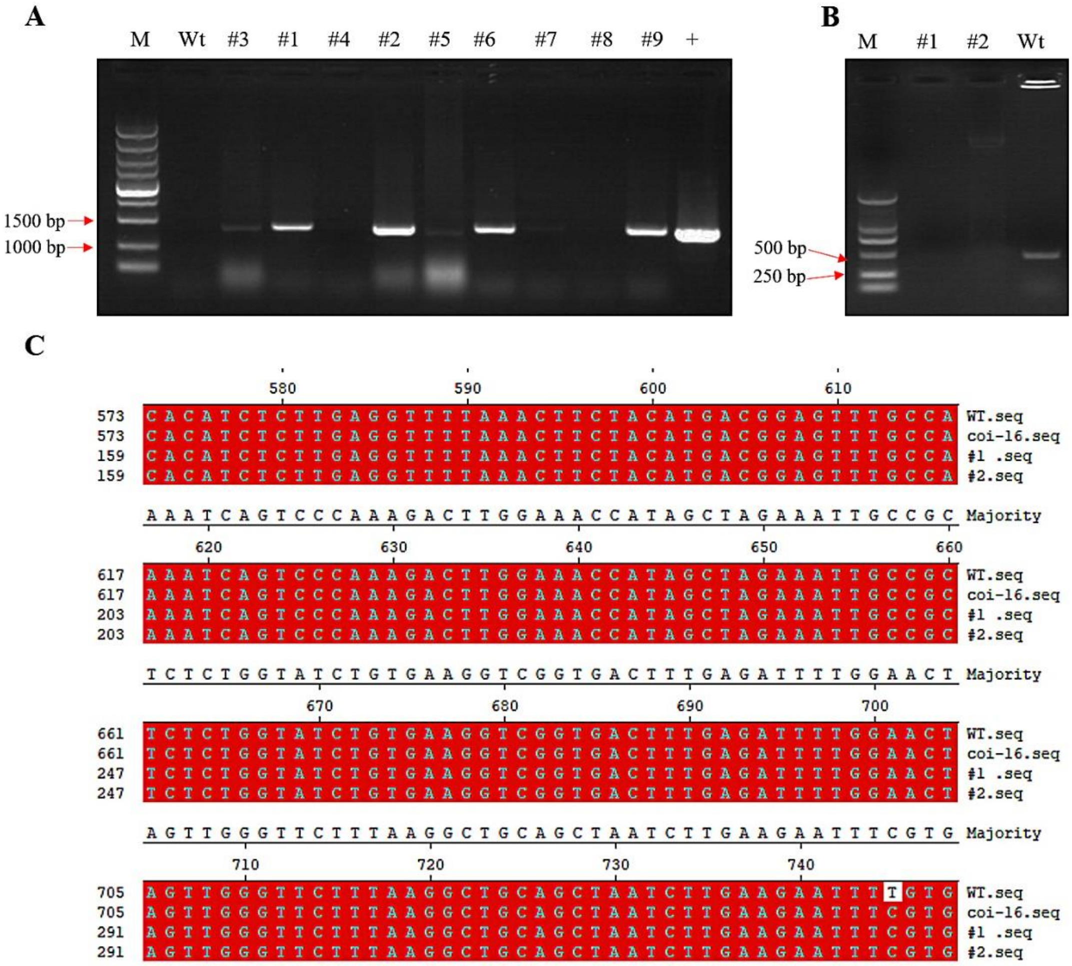

Figure S2. Verification of transgenic lines, the amplified product was 1280 bp (A); verification of *coi1-16* allele in #1, #2 (B). The primers were designed based on the SNP of COI1 in Wt and *coi1-16*. There was no product (350 bp) in homozygous background of *coi1-16*; alignment of sequencing results of Wt, *coi1-16*, #1, and #2 (C).

Table S1. The primers used for constructs and qPCR in the present study

Primer name	Sequence(5′-3′)
Com-*AtCOI1pro*-F	atttgaaaaatctcagaattcTTTTTTTGGTTCTTTGTAAGTGTGG
Com-*AtCOI1pro*-R	cgatcatccatCGGATCGAAAAAAAAACAAAAGA
Com-*AaCOI1*-F	ttcgatccgATGGATGATCGAACACCATCAAA
Com-*AaCOI1*-R	ggtcttaattaactctctagaTTATGGAGCAACGGAGACTAGTGG
Sub-*AaCOI1-F*	ttacatttacaattaccatggATGGATGATCGAACACCATCAAA
Sub-*AaCOI1-R*	ttgctcaccatTGGAGCAACGGAGACTAGTGG
Sub-*AaCOI1-GFP*-F	gttgctccaATGGTGAGCAAGGGCGAGG
Sub-*AaCOI1-GFP*-R	ggtcttaattaactctctagaTTACTTGTACAGCTCGTCCATGCC
q-*AaACTIN*-F	CCCCTGCTATGTATGTTGCCA
q-*AaACTIN*-R	CGCTCGGTAAGGATCTTCATCA
q-*AaCOI1*-F	AAATGCACTTGCACTGGCTG
q-*AaCOI1*-R	GAGCAACGGAGACTAGTGGG
q-*AtPDF1.2*-F	TCATGGCTAAGTTTGCTTCC
q-*AtPDF1.2*-R	AATACACACGATTTAGCACC
q-*AtVSP1*-F	GGAGACCTTGCATCTATACGAA
q-*AtVSP1*-R	CGTTTGGCTTGAGTATGAGATG
q-*AtVSP2*-F	CAAAGGACTTGCCCTAAAGAAC
q-*AtVSP2*-R	GTCTTCTCTGTTCCGTATCCAT
q-*AtGAPDH*-F	TCCGGAACTCAATGGAAAACTA
q-*AtGAPDH*-R	ACAAGTTTCATGAAGGACTTGC

Table S2. Cis-elements identified in the promoter of AaCOI1.

Site name	Organism	Position	Sequence	Function
AE-box	*Arabidopsis thaliana*	647	AGAAACTT	part of a module for light response
chs-CMA2a	*Petroselinum crispum*	1103	TCACTTGA	part of a light responsive element
GT1-motif	*Avena sativa*	1311	GGTTAAT	light responsive element
chs-CMA1a	*Daucus carota*	-611	TTACTTAA	part of a light responsive element
MYC	*Arabidopsis thaliana*	247, 393	CATGTG	
CTAG-motif	*Avena sativa*	362,	ACTAGCAGAA	
O2-site	*Zea mays*	838, 876, 942	GATGA	cis-acting regulatory element involved in zein metabolism regulation
STRE	*Arabidopsis thaliana*	1716	AGGGG	
MYB	*Arabidopsis thaliana*	1495, 1778, 1946	CAACCA	
MBS	*Arabidopsis thaliana*	-1044	CAACTG	MYB binding site involved in drought-inducibility
TCA	*Pisum sativum*	-378	TCATCTTCAT	
CCGTCC motif	*Nicotiana tabacum*	-916	CCGTCC	
TGACG-motif	*Hordeum vulgare*	949, 1599	TGACG	cis-acting regulatory element involved in the MeJA-responsiveness
CAAT-box	*Nicotiana glutinosa*	122, 270, 226	CAAT	
CAAT-box	*Pisum sativum*	-465, -473, 1370	CAAAT	common cis-acting element in promoter and enhancer regions
CAAT-box	*Arabidopsis thaliana*	-927, -1092, 1498	CCAAT	common cis-acting element in promoter and enhancer regions
TCT-motif	*Arabidopsis thaliana*	-644, -863	TCTTAC	part of a light responsive element
CGTCA-motif	*Hordeum vulgare*	-949, -1599	CGTCA	cis-acting regulatory element involved in the MeJA-responsiveness
Myb	*Arabidopsis thaliana*	29, 60, 196	TAACTG	
AT-TATA-box	*Arabidopsis thaliana*	769	TATATA	
TC-rich repeats	*Nicotiana tabacum*	1115	ATTCTCTAAC	cis-acting element involved in defense and stress responsiveness
as-1	*Arabidopsis thaliana*	949, 1599	TGACG	
TATA-box	*Arabidopsis thaliana*	-409, 410, 769	TATA	core promoter element around -30 of transcription start
TATA-box	*Brassica napus*	408, 768, 770	ATATAT	core promoter element around -30 of transcription start
TATA-box	*Helianthus annuus*	-1363, -1751	TATACA / TATAAA	core promoter element around -30 of transcription start

TATA-box	*Brassica oleracea*	1681, 1628	ATATAA	core promoter element around -30 of transcription start
TATA-box	*Pisum sativum*	-1750,	TATAAAA	core promoter element around -30 of transcription start
Box 4	*Petroselinum crispum*	826	ATTAAT	part of a conserved DNA module involved in light responsiveness
A-box	*Petroselinum crispum*	-916	CCGTCC	cis-acting regulatory element
CCGTCC-box	*Petroselinum crispum*	-916	CCGTCC	
HD-Zip 3	*Arabidopsis thaliana*	-1939	GTAAT(G/C)ATTAC	protein binding site

Data S1. Amino acid sequence of AaCOI1:
>PWA88385（AaCOI1）
MDDRTPSNQRLHGMSDVVFECVLPYIHDSRDRQSISLVCKRLYELEAQTRKHVTIALCYTSTPK
QLLQRFPYLESLKLKGKPRAAMYNLIPEDWGGFATPWVEELAKSFKCLKSVHFRRMIVKDSDL
ELLARERGHVLQALKLDKCSGFSTDGLLHICRSSRNLKILFFEESQVIEKDGEWLHELAMNNT
VLETLNFYMTDLSQVSFKDLELIAKRCKSLVSVKIGDCEILDLVGFFKSAVALEEFSGGCFNHQA
EQYASVVYPPRLCRLGLNYMSTNEMPFVFPFASRLKKLDLLYALLDTDDHCLLLQRCPNLEVL
ETRNVIGDRGMEVLGSCCRKIKRLRIERGADEQEMEDEEGVVSQRGLTAIAQGCLDLEYIAVY
VSDITNAALETMGKNLTNLCDFRMVLLDKEEVITDLPLDNGIRSLLSGCNKLRRFALYLRPGGL
TDVGLSYIGQYSQNIRWMLLGYLGESDSGLLGFSTGCPSLQKLEVRGCCFSENALALAVLQLKS
LRYLWVQGYRGSRTGCDLLAMARPYWNIEIIPPRKVNLGEGRETEHPAHILAYYSLAGPRTDF
PPSVIPLVSVAP

Data S2. Native promoter of *AtCOI1*
TTTTTTTGGTTCTTTGTAAGTGTGGTCCGAGTTAAGTGTATAGAAAACTCATCTCTCGCCTA
GTTTTTGGTTTTGTTCAGCCCAAGTCTTTTGGTTTCAGGTGTAATTAGTCGGGTTTTTTGTA
GCTTGCTTATATTACCTTTGTTATTTCCGAGTTTCAAATGAAGCTAAGAAATACGATCATAA
TAAAAAAGTTCTTTTGCAGTATTCATTTTCTTTCATGGTTCCCTCCTTATTCATAGTCATTGT
TGTTCTTTCTAATACTTTTCCTGAGCTTTTTTTTTCTATTGGAAAAACTTTTGTCACTGAAAG
AAATTTCAATGTGATTGGTTCTTTTCGCAACTGTAATAAGCACTAAAAGTTAATAATTTGC
TGCTTTTTAAGTCTTGTTGCTGAATGATTTAATCCCAAAGACGTTAAAAAAAAGACATGTA
AATAGTCTTTGTTCCAAAAGTACCATTTTGAGCGCTTTCTTTCGACAATTGATGTTGAACT
TGTACCAAATAAACCTTGAAAATCATACATCATTAAATGTAGTTTTTTGTTATATACTTATAT
TCATATGGTCCATAATATATAGTTAACCATATTATGGACCATATAAATTCATGCAGTCAACA
ACTTTTTTTTTCCTCATTAAAAGCTACTAATACTGTAGAAAATTTTTATCTTACAAAAGAAA
GGAGAATTATTGTATTTTTATTTTAATCACATATAAAGCAACCAAAGATAACATATACTAGT
ATTTAGTATTTTTAAATTTTTTGCTTAGGATTCAGGTTATTAATACTCTATAAAGAAATAATT
AAATCTATCCATCGAATAAATCACACAGCTTATTGGATCAGTTAAATATTCTAATAATATTG
TCGTGTAGCTGAGATCTGACCACTGCAAAAATGAAAAGAAAAACATAGAAGTAGAGAG
AAGATCGCATCTCGACCGTCAACTTCAGTGTATGAAATAATGATCGTCCCACTTGATCCTC

AAAAATATTATTAACCAAACAAAATTTGATTCCATCGTCCCACTTTCTTCTTCTTCCTCCCA
ATCCGCCTCTTCTTCCTACGCGTGTCTTCTTCTCCCTCACTCTCTCAATCTCTAGTCTTCTCC
GATTCACCGGATCTTTCCTTTCTTACTTCTTTCTTCTCACTCTGGTGGTTATGTGTGGATCTG
CGACCTCGATTTCAATTCGAAGTCGTCGGTTTCTTCTCTAAATCGAATCTTTCCAGGATTC
GTTTGTTTTTTTCTTTTGTTTTTTTTTCGATCCG

Data S3. ***AaCOI1*** **CDS**
ATGGATGATCGAACACCATCAAACCAACGGCTTCACGGCATGTCAGACGTGGTATTCGA
ATGCGTGTTACCATACATACACGACTCACGTGACCGGCAATCAATCTCACTAGTCTGCAA
ACGTTTATACGAACTCGAAGCACAGACTCGTAAACACGTAACAATAGCATTATGTTACAC
CTCAACTCCTAAGCAGTTGTTGCAGAGGTTTCCTTATTTAGAATCCTTGAAACTGAAGGG
AAAGCCAAGGGCTGCGATGTATAACTTGATTCCTGAAGATTGGGGAGGTTTCGCGACTCC
GTGGGTTGAAGAGCTTGCGAAATCTTTTAAGTGTTTGAAAAGTGTTCATTTTAGAAGAAT
GATTGTGAAGGATTCTGATCTTGAGTTGTTGGCACGTGAGAGAGGTCATGTGCTTCAAGC
TCTTAAGCTTGATAAGTGTTCTGGTTTTAGTACTGATGGCCTTTTGCATATTTGTCGATCTA
GCAGGAATCTCAAAATCTTGTTTTTCGAAGAAAGCCAAGTAATAGAGAAAGATGGAGAG
TGGTTACATGAGCTTGCTATGAACAATACGGTACTTGAAACATTGAACTTTTACATGACA
GATCTTTCACAAGTTAGCTTTAAAGATCTTGAGCTAATAGCCAAAAGATGCAAATCTTTA
GTATCGGTAAAGATAGGAGATTGTGAAATCTTGGATCTTGTTGGTTTCTTCAAATCTGCTG
TCGCACTTGAAGAATTCAGTGGTGGTTGCTTTAATCATCAAGCAGAGCAGTATGCTTCTGT
GGTGTATCCCCCTAGATTGTGTCGTTTAGGTCTGAATTACATGAGTACAAATGAAATGCCC
TTTGTGTTCCCCTTTGCTTCCCGCCTGAAAAAACTGGATCTCCTATATGCCCTTCTTGACAC
TGATGACCATTGTCTGTTGCTCCAACGGTGTCCCAACTTGGAAGTACTCGAGACAAGAA
ATGTGATTGGTGACCGGGGAATGGAAGTTCTGGGGAGTTGCTGTAGAAAAATAAAGCGG
CTTAGGATTGAGAGAGGAGCCGATGAACAAGAAATGGAAGATGAAGAAGGTGTTGTTT
CTCAAAGGGGGTTGACCGCCATAGCACAAGGTTGTCTTGATTTGGAATACATTGCTGTAT
ACGTATCTGATATCACAAACGCAGCTTTAGAAACCATGGGCAAGAACTTGACAAACCTT
TGTGATTTCCGTATGGTTTTGCTGGATAAAGAAGAAGTTATCACAGACTTGCCTCTCGACA
ATGGAATCCGATCTTTGTTAAGTGGCTGCAATAAACTTAGAAGATTTGCACTCTATCTTAG
ACCAGGAGGGCTGACGGATGTGGGTTTGAGTTACATCGGTCAATATAGCCAGAATATAAG
ATGGATGCTACTCGGGTATCTTGGAGAATCTGATTCCGGGCTTCTAGGCTTCTCCACAGGC
TGCCCGAGCCTACAAAAACTTGAAGTAAGAGGGTGTTGTTTCAGTGAAAATGCACTTGC
ACTGGCTGTTCTCCAGCTGAAGTCTTTAAGATACTTATGGGTACAAGGGTATAGGGGATC
AAGGACTGGATGTGATCTGTTAGCAATGGCGCGCCCATATTGGAATATTGAAATAATTCCT
CCAAGAAAGGTTAATCTTGGGGAAGGTAGAGAGACAGAACATCCTGCACATATACTTGC
ATATTACTCTCTTGCTGGACCAAGAACCGATTTTCCACCTTCTGTCATCCCACTAGTCTCC
GTTGCTCCATAA

Data S4. The 2000bp sequence designated as the promoter of ***AaCOI1***
GTACATATTATCGAAAAAACTGAGGTGCTAGCTAGTGTAAAAATGCTTGTGGTTATCATTA
CTAAAATATGATGATCTCAAATTTAGTTCTTGCTAGTTGCAACAACGTTGAGGAATTCACC
TCGCCAAACTTCTGATAACAGAAAATTTTACCCGATTTGAATATGTGTTTAGATGGATTAA

CACCGAGGAATAGTCAGGCGGAAAACAAAAGCCATGGTTGCGCAAAAATAAAGTTACT
TATTATAAAAAGAAATAAATGTGATATATGAGATTATTAAGGGGCGTGTGTTTAAGCTTATT
TGATTTCCTGCTTATATTTCACAATCTTAAATTTTTCTGCTTGCTTATTCTGATATTTGTAGGT
TTATATGAGTTTATTATGATATACTCCCTCCGTCAGTGTTCACTTTAAAAGTGCACAAAGTT
TTAAAAAATTTCATTAACTACACTCTCTATCAATCAAATATCATTTTTACCCACTAAAGTCT
CTATCTCATTGGTTGGAATAGAATATGTACACTTAATTTGAGACATCCAAAGATTCCATCG
TGGACATTTAATATGAGACGAAAGGAGTAATAAGCTTAACATAAGCTCATAAATTTCAAA
ATAAGTCAAAAATTCAAATTTATACAGTAAAATATAAGATGAAATCAGATAAACTTATTTT
ACCGGGGTGAATTAACCCTTTTACCAAAAAAAAAAAAATCAGATAAACTCATAAATAAGT
TTAAACAAACATACTTAATACCGTGTACAATCATTTTCTACTACACTTAATTAGCCAGTAA
AAGTAAATCTAGTTAAAAAAAAAAAAAAAAAAAAAAAAAAAAATAGTTCCAAAGACACA
AGAGTATCTATGTAGTTTAATGAAGAGTTAGAGAATCACATCAAGTGAGTTCCCCCAATT
AGCCATTGACACACCTCCATTAAATACCTCCTCCTCACTCACAACTGTCTTTCTTAAAGTT
GTTCTTGGCTGCAGGACCTTTTTAATCAAACCTTTCTATTTTTATCGGTTATCATACATACTC
ATCCATCTGTCACCGTCAGATCATCACACCATCATCCAATCTCAGCCGTCCATTCTCTCTC
TAGCCCTCCATTCGTGGATCTCATCTCATCCCTTCACTCTTACATTCATCATATCTTTTCATT
TCATCCACAATATTAATACTGTAATAATCTCTCATAAATCACTCTTCTTTTTATACTAATCCC
TTTCATATATATCTTTAATAATCTTTCTTGATTTCAAGTTGATTTTTTCTTTTTTACTTTTTTCA
ACACTTTCCTGATTTAAACTTCAAGATTTTGTTTCAGAAATCAATAAAAATAAAAAAAAA
AAGTTTCTTACTTTCAACTTGGGTTTTTCATTTTTGTTACTTAATTGATTCCAACTTCTTCAC
ATATCATAAATCTGTTTTCTATTCTTTGATTTTTAAGTTCAGAGGCCAGTCTAAGTAATCAA
AAGTTTGTCAATTTCAGCTTGAGGTATTGCTACTTTCCTAAAATAGAAGTTCCAAATCTTC
AAATCAAGAATCTTTAATTCTGATTTAATCTTGATTTCAGCTTCTGGGTATTGTTTATAATCC
ATAATCTCACATGGGTTTTCATTTTCATACCATTTTCTGTTAGTTTCAGTTAGTTTTCAGTTA
ATTCTGTTATTTTCTTGAATTTTAAGGTTCTTCTTCAGTTTTGCTTAAATCTTGATTTCACTTC
TGGGTATTGTTTAGAATTCATAATCTCACATGGGTTTTCATTTTCAATTCAATTATCTGTTAA
TTTCAGTTAGTTTTCAGTTAATTCTGTTATTTTCTTGAATTTCAAGGGTTTTCTTTAATTTTGC
TTAAATTTTGATTTCAACTTCTGGGTATTGTTTAGAAACTAAAAAGATTCATAATCTCATCT
GGGTTTTCATTTTACCGTTATTTTCAGTTAAATTTCTGTTAATTCTGTTAAATTTCAGTTAATT
CTGTTATTTTCTTGAAGTTGATCAT
